# Electrocardiograms from different types of exercise in Eventing horses with and without cardiac signs

**DOI:** 10.1111/evj.14449

**Published:** 2024-12-04

**Authors:** Cristobal Navas de Solis, Claire Solomon, Mary Durando, Darko Stefanovski

**Affiliations:** ^1^ Large Animal Clinical Sciences, School of Veterinary Medicine University of Pennsylvania Philadelphia Pennsylvania USA; ^2^ Equine Sports Medicine Consultants Newark Delaware USA

**Keywords:** arrhythmia, atrial fibrillation, equestrian sports, horse, premature complex

## Abstract

**Background:**

Exercising arrhythmias can be clinically irrelevant or associated with poor performance, collapse and sudden cardiac death.

**Objectives:**

To test if readable exercising ECGs can be recorded by grooms or riders and to describe arrhythmias in ECGs from different types of exercise in Eventing horses and investigate associations with type of workout, the presence of previous cardiac signs and intensity of exercise.

**Study design:**

Cohort study.

**Methods:**

Single lead exercising ECGs were obtained by riders or grooms during training and competition from a convenience sample of horses in training for Eventing competitions. Arrhythmias were described, and associations between different arrhythmia categories and variables that described the horse and the workouts were sought.

**Results:**

There were 1002 ECGs from 62 horses (median [range] 7 [2–97] ECGs/horse) evaluated and 737 workouts (73.6%) were >95% readable and included in the analysis. There were arrhythmias in 250 (33.9%) of the workouts, complex arrhythmias in 13 (1.8%) and the number of premature complexes was median (range) 0 (0–118). Peak heart rate and duration of exercise were associated with the number of premature complexes, the presence of arrhythmias and complex arrhythmias and were colinear with the type of exercise. Having previous signs of cardiac disease and the type of workout were associated with higher odds of having arrhythmias.

**Conclusions:**

Monitoring the rhythm of equine athletes with ECGs obtained by riders and transmitted to an online cloud was feasible. Arrhythmias were frequent, and complex arrhythmias were rare. The presence of cardiac signs, type of exercise and peak heart rate were associated with the presence of arrhythmias. None of the horses developed poor performance or collapse attributed to cardiac disease. The arrhythmias that should be concerning for equine veterinarians need further definition.

## 1 INTRODUCTION

Exercising arrhythmias occur frequently in horses participating in all equestrian disciplines.^1^, ^2^, ^3^, ^4^, ^5^, ^6^, ^7^ In most instances exercising arrhythmias occur in the absence of clinical signs and are common in horses without signs of cardiac disease.^1^, ^2^, ^3^, ^6^, ^7^ The association between arrhythmias and performance is unclear, and many successful horses display arrhythmias during and after exercise.^6^, ^8^ The intensity of the exercise, often estimated by the peak heart rate during exercise, has been shown to be a factor in the occurrence of arrhythmias during exercise,^1^, ^7^ and there are suggestions that the familiarity of the horse with the exercise performed during the ECG recording affects the frequency of arrhythmias.^8^, ^9^ The ability to analyse large sets of exercising electrocardiograms (ECGs) could help answer important questions about the relevance of exercising arrhythmias in the performance and safety of horses.

The importance of exercising arrhythmias is considered differently when underlying cardiac disease is present.^5^, ^10^, ^11^ Exercising ECGs have been recorded in horses with and without cardiac disease, but rarely have these horses been compared or monitored longitudinally.^6^, ^8^, ^9^, ^12^ Obtaining readable ECGs during exercise is time‐consuming, limiting its widespread use. Integrating ECG recordings into exercise routines without the on‐site presence of a veterinarian using fitness trackers and mobile health tools could allow frequent longitudinal monitoring of horses in training. The incidence of exercise associated sudden death in horses is over 200‐fold higher than in human beings^13^ and a relevant problem for the health and the welfare of horses and the social licence to operate in equestrian sports.^14^ However, many exercising arrhythmias do not lead to poor performance, and sudden death due to cardiac arrhythmias is uncommon in absolute terms.^1^, ^2^, ^3^, ^4^, ^5^, ^6^, ^7^, ^8^, ^9^, ^12^ For the use of exercising ECGs as a prevention tool, collection and analysis must have a feasible workflow for the equestrian industry.

The objectives of this study were: (1) to describe the feasibility of obtaining exercising ECGs using a fitness tracker by operators without veterinary medical training (riders and grooms); (2) to describe arrhythmias in eventers with and without previous cardiac signs and the occurrence in different types of workouts; and (3) to investigate if previous cardiac signs, the type of workout and heart rate during exercise are factors associated with the presence of arrhythmias. We hypothesised that horses with previous signs of cardiac disease, a higher heart rate during exercise and more demanding workouts would be associated with arrhythmia development.

## 2 MATERIALS AND METHODS

A cohort study with convenience sampling of 62 horses was used to longitudinally monitor Eventing horses in active training and competition between June 2020 and February 2024. Horses were ridden following their usual routines and monitored using fitness tracking (wearable) devices. Fitness trackers were fitted by the rider or groom, and the data was transmitted to an online cloud via a mobile device (after October 2021) or downloaded from the device onto a computer (before October 2021). Horses were recruited by investigators and collaborating veterinarians. Their veterinarian assessed horses as fit to exercise at the time of enrolment. Fit to exercise was defined as horses that, in the opinion of the veterinarian, were free of conditions that would affect the well‐being or markedly affect performance. Horses were included in this study while previously diagnosed conditions such as musculoskeletal, respiratory, or cardiac diseases that were subclinical were being managed or when a rehabilitation program had been completed. Examples of these diseases could be joint disease necessitating occasional anti‐inflammatory therapy, inflammatory airway disease, valvular disease, or arrhythmias that, at the judgement of the primary care veterinarian, were unlikely to affect safety, welfare, or markedly affect performance. Examples of these arrhythmias included second‐degree atrioventricular block, sinus arrhythmia, or occasional premature complexes without detectable underlying cardiac disease. Horses were divided into CARDIAC and NON‐CARDIAC groups. Horses were included in the CARDIAC group if they had previously presented to a veterinarian for signs that could be ascribed to the cardiovascular system, such as atrial fibrillation, valvular disease that was more than mild and myocardial diseases. All horses were considered safe to exercise by their veterinarians except for one horse for which it had been recommended that high‐intensity exercise was discontinued. This recommendation was made based on the presence of complex arrhythmias during exercise and myocardial hyperechoic areas. The rider declined to follow this recommendation and the horse was enrolled in the CARDIAC study group.

The fitness tracker (Arioneo Equimetre®, Paris, France) was used to collect exercising ECGs. The fitness tracker records a one‐lead ECG and data from GPS and motion sensors (accelerometer, gyroscope and magnetometer). The performance, accuracy and precision of the ECG and heart rate when using this device have been previously reported.^15^, ^16^ An online messaging application (WhatsApp®) was used to maintain communication between investigators, riders, grooms, primary care veterinarians and device and application developers. Conversations through the messaging application were used to troubleshoot issues with hardware or software, discuss results related to fitness tracking and provide information about basic principles of exercise physiology and equine sports medicine. The type of workout (TYPE) was classified as Flat, Hack, Trotting Set (Trot), Jumping Schooling (Jumping), Cross‐Country (XC) Schooling, Gallop and Cross‐Country Competition (Competition) following the common types of workouts used to describe exercise in Eventing horses. Horses were separated into levels based on the FEI level at which they were competing or the analogous USEF level. USEF levels that are less demanding than FEI 1* level were included in the 1* group. The time of the year (SEASON) was divided into three time periods: 1 = December–March, 2 = April–July, 3 = August–November according to the periods in which most horses have rest and build‐up periods (1), peak competition period (2) and late competition period (3).

**FIGURE 1:**
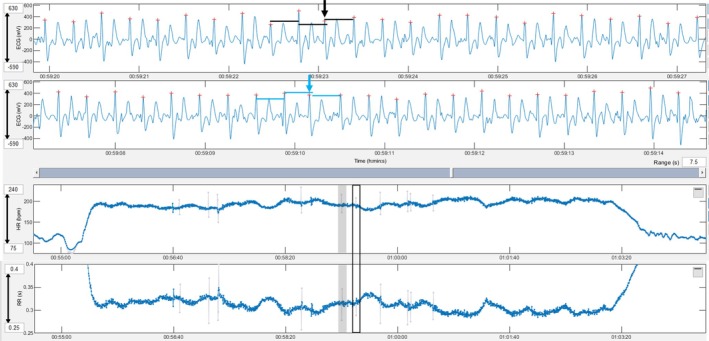
A premature narrow complex without a compensatory pause (black arrow) is shown in the top ECG and the box with a black border in the heart rate and RR tachograms. The black lines show the RR between the complexes immediately prior and after the PC being of the same duration. Note the shortening of the RR interval of the premature complex is not followed by prolongation of the subsequent RR. A premature narrow complex with a compensatory pause (blue arrow) is shown in the bottom ECG and the grey‐shaded area in the heart rate and RR tachograms. The blue lines show the QRS complex after the premature complex occurring after two times the RR interval before the premature complex. Note the shortening of the RR interval of the premature complex followed by an equal prolongation of the subsequent RR. Grey lines in the tachogram mark instantaneous heart rates that deviate more than 5% from the local average.

Electrocardiograms in which the quality allowed evaluation of QRS morphology and determination of RR intervals in 95% or more of the exercising recordings were considered readable and used for this study. Electrocardiograms were evaluated manually, and automated QRS markings were corrected. RR intervals that deviated 5% or more from the surrounding R‐R average were used to determine the presence of premature complexes (PCs) and were using ECG analysis tools and confirmed manually. A commercially available HRV software program (Kubios version 3.5 for Windows; Kubios Oy, Finland) was used for analysis. The beat correction setting was set at custom, and 5% of the RR at the peak heart rate of each recording was calculated. As the HRV software rounds the custom setting to the closest first decimal point, electronic callipers (https://stanford.edu/~rogersaj/) were used to determine if QRS complexes marked as premature at one decimal point and not at the next met the 5% prematurity criteria. Arrhythmia description followed a modification of criteria recently reported in cross‐country competition.^7^ Briefly, the presence of any type and number of premature complexes in a workout was defined as ‘ARRHYTHMIA’. The presence of couplets was defined as 2 consecutive premature complexes. The presence of complex arrhythmia (ARRHYTHMIA_complex_) was defined as the presence of triplets (3 consecutive premature complexes), tachycardia (>3 consecutive premature complexes), or paroxysmal atrial fibrillation. The nPC was defined as the number of all premature complexes (i.e., a triplet is defined as 3 nPCs). The presence of arrhythmias during fast heart rate deceleration at any time during exercise was defined as ‘ARRHYTHMIA_deceleration_’ and analysed separately due to the potential of arrhythmias during fast heart rate deceleration involving specific mechanisms related to autonomic balance.^2^ These categories were used as a dichotomous variables to classify workouts, and the number of premature complexes (nPC) per workout (during all parts of exercise) was analysed as a continuous variable to estimate the total arrhythmia burden. Premature complexes were described as wide or narrow based on subjective comparison with the appearance of the preceding QRS complexes. Due to the ECG system only recording one lead and because P waves were inconsistently visible at high heart rates, PCs were not further classified as ventricular or supraventricular. Single PCs were classified as having or not having a compensatory pause for descriptive purposes but not for statistical analysis. The pause category (compensatory or non‐compensatory) was reader‐determined and obtained using manual and digital callipers on the ECG trace and the R‐R tachogram to determine whether the coupling interval + return cycle length was equal or nearly equal to the sum of the previous R‐R intervals as previously described (Figure 1).^7^ The peak heart rate during exercise (HR_peak_) was the average rate of the 5 shortest RR intervals after excluding PCs and calculated by HRV software and was used as a surrogate of exercise intensity. The average heart rate from the initiation of exercise to the end was reported as HR_mean_ and calculated by HRV software.

### 2.1 Data analysis

All analyses were conducted with statistical software (Stata 17MP, StataCorp, College Station TX, USA) using two‐sided tests of hypotheses and a *p*‐value <0.05 as the criterion for statistical significance. Descriptive analyses included computation of medians and ranges of continuous variables and percentages, frequency counts and tabulation of categorical variables. Tests of normal distribution (Shapiro–Wilk) were performed to determine the extent of skewness of continuous variables. Initial exploratory univariate analysis was conducted using Spearman rank correlation. Negative values of *ρ* indicated inverse correlation. Rho was interpreted as defined by Chan (None = 0, Poor = [0–0.2], Fair = [0.2–0.6], Moderate = [0.6–0.8], Strong = [0.8–1], Perfect = 1).^17^

Inference statistical analysis was based on a multivariable mixed‐effects logistic regression model to assess the likelihood of ARRHYTHMIA, ARRHYTHMIA_deceleration_ and ARRHYTHMIA_complex_ and multivariable mixed‐effect Poisson regression model was used for the assessment of risk for nPCs in a similar fashion. The presence of previous signs of cardiac disease (CARDIAC group vs. NON‐CARDIAC group) and the TYPE of workout were set as the categorical fixed effects of the model. The model was confounded by age (years), level (1*–5*), SEASON (1–3), duration (minutes) and HR_peak_ and random effects set at the level of the individual animal. The associations between the binary outcome of interest and independent variables of the model from the mixed‐effects linear regression model were reported as odds ratio (OR) with their respective 95% confidence interval (95% CI). The Poisson regression associations between the outcome of interest and the independent variable of the model were reported as an incidence rate ratio (IRR) with their respective 95% CI.

## 3 RESULTS

A total of 1002 ECGs with a duration of (median [range]) 50 [15–202] minutes were available for review. These recordings were obtained from 62 horses that were 11 [6–19] years of age. Fifty‐five horses were Warmbloods, and 7 were Thoroughbreds. There were 55 geldings, 5 mares and 2 stallions. There were, on average, 7 workouts/horse with a range of 2–97 workouts per horse, and these were from (horses/workouts) 15/171 1* level, 13/308 2* level, 12/203 3* level, 12/162 4* level and 10/158 5* level. Two hundred and seventy‐four recordings from 8 horses were in the CARDIAC group and 728 recordings from 54 horses were in the NON‐CARDIAC group. Horses were included in the CARDIAC group due to persistent atrial fibrillation that was cardioverted (*n* = 4), poor performance due to an episode of paroxysmal atrial fibrillation and myocardial injury (*n* = 1), acute onset of a murmur associated with a nodular lesion in the tricuspid valve (*n* = 1), complex arrhythmias and multiple hyperechoic regions in the myocardium (*n* = 1) and collapse during exercise (*n* = 1).

The % of readable trace within each recording was 100 [0–100]%. Seven hundred and thirty‐seven (73.6%) exercising ECGs were 95% readable or more. When TYPE of workout was assessed for readability using Flat workouts as the reference, Trot, Jumping, Gallop and Competition recordings were more likely to be readable (Table 1).

**TABLE 1:** Number of recordings (*n*) in each type of workout (TYPE), percentage (%) of readable recordings for each type or workout and OR (CI) and p values for workouts of each TYPE being readable (>95% readable) using ‘Flat’ workouts as a reference.

TYPE	*n*	% readable recordings	OR (95% CI)	*p*
Flat	307	68.1%	Reference (1)	NA
Hack	128	60.2%	0.7 [0.44–1.2]	0.2
Trot	41	87.8%	3.8 [1.4–10.5]	0.009
Jumping	129	79.8%	1.7 [1.02–2.9]	0.04
XC School	46	78.3%	1.84 [0.86–4.1]	0.1
Gallop	324	77.5%	1.9 [1.3–2.9]	0.002
Competition	27	92.6%	4.5 [0.96–20.9]	0.06
Total	1002	73.6%	NA	NA

**FIGURE 2:**
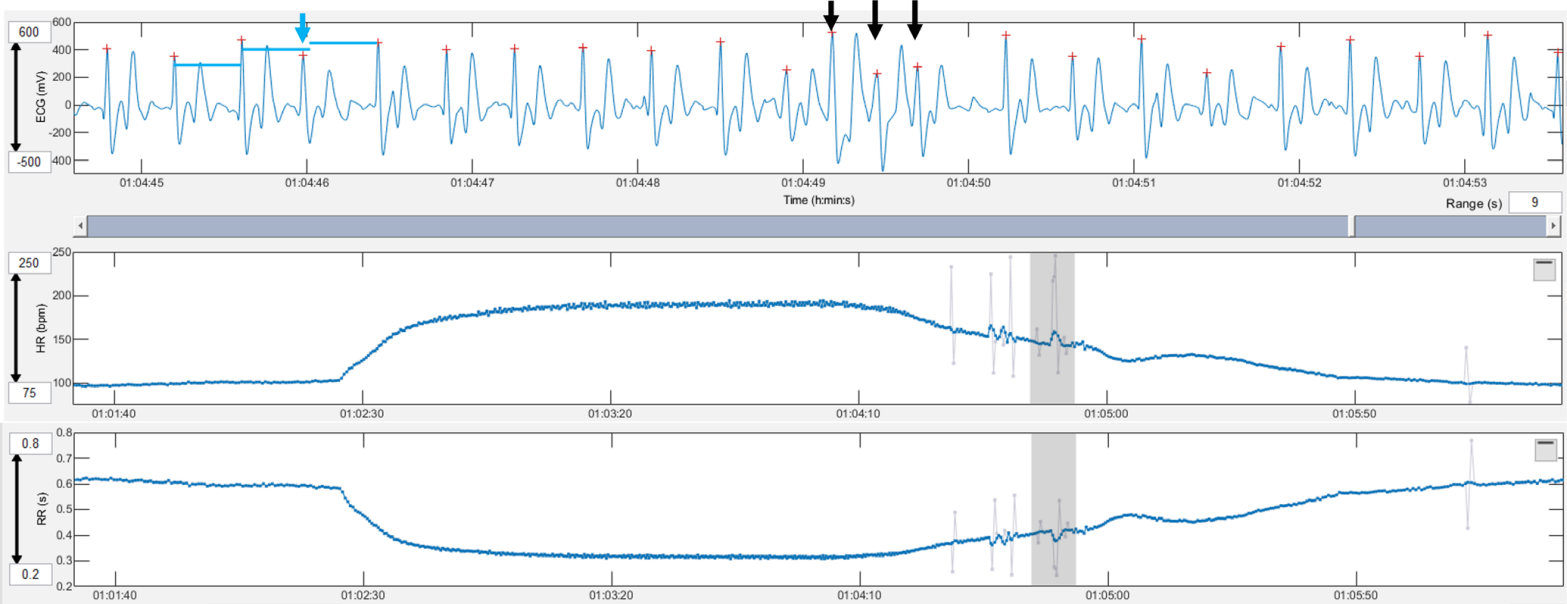
A premature narrow complex with a compensatory pause (blue arrow and blue lines) and triplet of premature narrow complexes (black arrows) in the heart rate deceleration period are shown. Grey lines in the tachogram mark instantaneous heart rates that deviate more than 5% from the local average.

**FIGURE 3:**
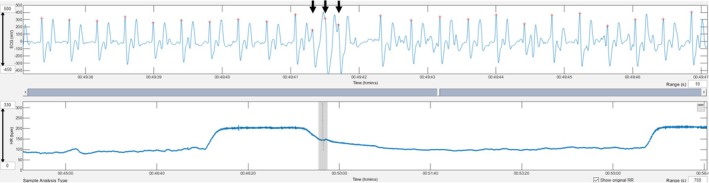
A triplet of premature wide complexes during deceleration (black arrows) is shown. Grey lines in the tachogram mark instantaneous heart rates that deviate more than 5% from the local average.

**FIGURE 4:**
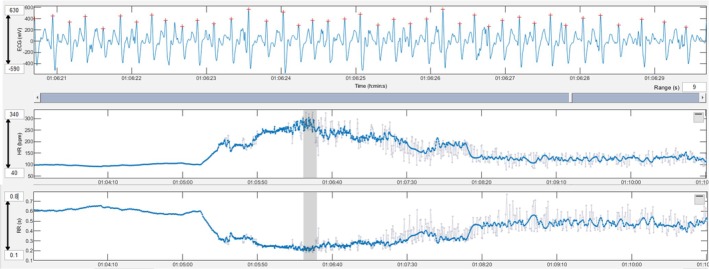
Paroxysmal atrial fibrillation developed during exercise. An irregularly irregular rhythm with variable RR intervals can be seen in the ECG and the heart rate tachogram. Grey lines in the tachogram mark instantaneous heart rates that deviate more than 5% from the local average.

The summary of the arrhythmias and heart rates identified in different TYPE of horses' workouts in the CARDIAC and NON‐CARDIAC groups are summarised in Table 2 and the description of the arrhythmia is included in Table 3. The 13 workouts (15 arrhythmia instances) of ARRHYTHMIA_complex_ are described in Box 1. Figures 1, 2, 3, 4 show examples of arrhythmias identified in horses in the study.

**TABLE 2:** Summary of arrhythmia categories and heart rates identified in different types of workouts (TYPE) of horses in the CARDIAC and NON‐CARDIAC groups.

	TYPE	*n*	nPC	ARRHYTHMIA	ARRHYTHMIA deceleration	ARRHYTHMIAcomplex	HRpeak (/min)	HRmean (/min)
CARDIAC	Flat	41	0 [0–14]	13 (31.7%)	3 (7.3%)	0	141 [102–202]	77 [56–107]
	Hack	42	0.5 [0–114]	21 (50%)	9 (21.4%)	0	141.5 [94–211]	76 [56–105]
	Trot	7	0 [0–10]	1	1	0	148 [96–194]	82 [64–98]
	Jumping	29	0 [0–11]	14 (48.3%)	4 (13.8%)	1 (3.4%)	177 [124–209]	95 [65–122]
	XC school	7	2 [0–10]	5	4	0	195 [169–206]	99 [67–122]
	Gallop	60	2 [2–21]	43 (71.7%)	13 (21.7%)	4 (6.7%)	206 [143–230]	111 [87–144]
	Competition	16	2.5 [0–18]	12	2	0	211 [195–226]	108 [85–137]
	Total	202	1 [0–114]	109 (54%)	35 (17.3%)	5 (2.5%)	180 [94–230]	93 [56–144]
NON‐CARDIAC	Flat	168	0 [0–12]	16 (9.5%)	2 (1.2%)	0	138.5 [98–205]	87 [68–125]
	Hack	35	0 [0–7]	6 (17.1%)	1 (2.9%)	0	147 [84–196]	81 [55–121]
	Trot	29	0 [0–36]	5 (17.2%)	2 (6.9%)	0	145 [107–205]	92 [59–125]
	Jumping	74	0 [0–118]	21 (28.4%)	9 (12.2%)	0	176 [125–218]	95 [71–131]
	XC school	29	0 [0–2]	6 (20.7%)	2 (6.9%)	0	192 [173–209]	110 [84–134]
	Gallop	191	0 [0–101]	81 (42.4%)	31 (16.2%)	8 (4.1%)	192 [147–240]	100.5 [63–154]
	Competition	9	2 [0–17]	6	3	0	215 [198–218]	122 [107–147]
	Total	535	0 [0–118]	141 (26.3%)	50 (9.3%)	8 (1.5%)	173 [143–240]	94.5 [55–154]

*Note*: *n* = number of recordings, nPC = number of premature complexes. ARRHYTHMIA = workouts with arrhythmia of any category present, ARRHYTHMIA_deceleration_ = workouts with arrhythmias present during fast heart rate deceleration at any time during exercise. ARRHYTHMIA_complex_ = workouts with complex arrhythmias. nPC, HR_peak_ and HR_mean_ are described as median [range]. Arrhythmia categories are described as the number and percentage (in parenthesis) of readable recordings (for each workout indicated in the table's line) with the type of arrhythmia present.

**TABLE 3:** Summary of arrhythmia descriptions identified in different types of workouts (TYPE) of horses in the CARDIAC and NON‐CARDIAC groups.

	TYPE	*n*	NARROW WITH_exercise_	NARROW WITHOUT_exercise_	WIDE WITH_exercise_	NARROW WITH_deceleration_	NARROW WITHOUT_deceleration_	WIDE WITH_deceleration_	COUPLET_exercise_	COUPLET_deceleration_	COMPLEX_exercise_	COMPLEX_deceleration_
CARDIAC	Flat	41	0 [0–13]	0 [0–6]	0	0 [0–5]	0	0	0	0	0	0
	Hack	42	0 [0–108]	0 [0–2]	0	0 [0–5]	0 [0–3]	0	0 [0–3]	0	0	0
	Trot	7	0	0	0	0 [0–10]	0	0	0	0	0	0
	Jumping	29	0 [0–5]	0 [0–1]	0 [0–5]	0 [0–6]	0	0 [0–1]	0 [0–1]	0	0 [0–1]	0
	XC school	7	0 [0–10]	0 [0–2]	0 [0–3]	0 [0–1]	0 [0–1]	0	0	0	0	0
	Gallop	60	0 [0–15]	0 [0–3]	0 [0–2]	0 [0–3]	0 [0–4]	0	0 [0–3]	0 [0–1]	0 [0–2]	0
	Competition	16	2.5 [0–9]	0 [0–3]	0 [0–1]	0 [0–1]	0	0	0 [0–1]	0 [0–1]	0	0
	Total	202	0 [0–108]	0 [0–6]	0 [0–5]	0 [0–10]	0 [0–4]	0 [0–1]	0 [0–3]	0 [0–1]	0 [0–2]	0
NON CARDIAC	Flat	168	0 [0–12]	0 [0–1]	0 [0–2]	0 [0–1]	0 [0–1]	0	0	0	0	0
	Hack	35	0 [0–2]	0 [0–7]	0	0 [0–2]	0	0	0	0	0	0
	Trot	29	0 [0–36]	0 [0–2]	0	0	0	0	0	0	0	0
	Jumping	74	0 [0–118]	0 [0–1]	0	0 [0–10]	0	0 [0–1]	0	0 [0–1]	0	0
	XC school	29	0 [0–2]	0 [0–2]	0	0	0	0 [0–1]	0	0	0	0
	Gallop	191	0 [0–96]	0 [0–8]	0 [0–3]	0 [0–7]	0 [0–3]	0 [0–1]	0 [0–2]	0 [0–4]	0 [0–2]	0 [0–1]
	Competition	9	2 [0–6]	0 [0–5]	0	0 [0–1]	0	0	0 [0–1]	0 [0–3]	0	0
	Total	535	0 [0–118]	0 [0–8]	0 [0–3]	0 [0–10]	0 [0–3]	0 [0–1]	0 [0–2]	0 [0–4]	0 [0–2]	0 [0–1]

*Note*: Single nPC are divided according to their morphology (WIDE or NARROW) and according to the presence (WITH) or absence (WITHOUT) of a compensatory pause. The different categories are described using median [range]. Arrhythmias during periods of fast heart rate deceleration are reported separately under the subscript ‘deceleration’ and arrhythmias during any other portion of the exercise period different than fast heart rate deceleration are labelled with the subscript ‘exercise’.

BOX 1 **Description of the 13 workouts (15 arrhythmia instances) of complex arrhythmias.**

**Table:** 

Triplets of premature narrow complexes during exercise were observed in 8 workouts (9 arrhythmia instances). Five workouts were in one horse in the CARDIAC group (4 during gallops and 1 during jumping), and 3 workouts (1 each in 3 horses) were in the NON‐CARDIAC group (one workout had 2 instances). The horse in the cardiac group had hyperechoic areas in the myocardium. Triplets of premature narrow complexes in the fast heart rate deceleration period were observed after 2 workouts (Gallops) in 2 horses (one each) in the NON‐CARDIAC group (Figure 2). A triplet of premature wide complexes during fast heart rate deceleration was observed after 1 workout (Gallop) in a horse in the NON‐CARDIAC group 1 (Figure 3). Two episodes of paroxysmal atrial fibrillation were observed after 2 workouts (Gallops) from the same horse in the NON‐CARDIAC group (Figure 4). One run of 6 premature narrow complexes during exercise was observed in one workout (Gallop) in a horse in the CARDIAC group. This horse had a triplet of premature narrow complexes during the same workout. The horse in the cardiac group had hyperechoic areas in the myocardium.

Significant Spearman's rho (*ρ*) associating ARRHYTHMIA, ARRHYTHMIA_deceleration_, ARRHYTHMIA_complex_ with the cardiac group, TYPE, age, level, season and HR_peak_ are described in Table 4. No associations with SEASON were found, and this variable is not displayed in the table.

**TABLE 4:** Significant Spearman's rho (*ρ*) and *p* value associating ARRHYTHMIA, ARRHYTHMIA_deceleration_, ARRHYTHMIA_complex_ with cardiac group (CARDIAC), type of workout (TYPE), age, level, HRpeak and duration.

	Age	Level	CARDIAC	TYPE	HR_peak_	Duration
nPC	0.37 <0.001	0.20 <0.001	0.28 <0.001	0.06 0.1	0.36 <0.001	0.025 <0.001
ARRHYTHMIA	0.35 <0.001	0.17 <0.001	0.27 <0.001	0.06	0.36 <0.001	0.23 <0.001
ARRHYTHMIA_complex_	0.15 <0.001	0.11 0.003	NA	NA	0.15 <0.001	0.08 0.04
ARRHYTHMIA_deceleration_	0.17 <0.001	0.07 0.07	0.11 0.004	0.09 0.01	0.22 <0.001	0.11 0.002

*Note*: Rho was interpreted as defined by Chan YH (None = 0, Poor = [0–0.2], Fair = [0.2–0.6], Moderate = [0.6–0.8], Strong = [0.8–1], Perfect = 1).^17^ NA values could not be calculated due to amount of data available.

Belonging to the CARDIAC group (OR = 3.0 CI [1.1–8.3], *p* = 0.02) and the type of workout (Table 5) were associated with ARRYTHMIA. The type of workout (Table 5) but not the CARDIAC group was associated with ARRHYTHMIA_deceleration_. Belonging to the CARDIAC group (OR = 6.6 CI [2.9–15.1], *p* < 0.001) and the type of workout (Table 5) were associated with nPC. Logistic regression models could not be used to assess associations ARRHYTHMIA_complex_ due to the small number of complex arrhythmias in the data set. While HR_peak_ showed a significant association with the outcome of interest, ARRHYTHMIA, it had to be removed from the multivariable model due to collinearity with the type of exercise as a fixed effect. Similarly, duration and type of exercise could not be included in the same regression models as they were colinear. Age and level did not have an effect on the different arrhythmia categories when included in regression models.

**TABLE 5:** Results of mixed‐effects logistic regression assessing the likelihood of ARRHYTHMIA and ARRHYTHMIA_deceleration_ and mixed‐effect Poisson regression assessing the likelihood of nPCs.

	ARRHYTHMIA	ARRHYTHMIA_deceleration_	nPC
	OR [95% CI], *p*	OR [95% CI], *p*	IRR [95% CI], *p*
CARDIAC	3.0 [1.1–8.3], 0.03	2.65 [0.89–7.89], 0.08	6.6 [2.9–15.1], <0.001
TYPE			
Flat	Reference	Reference	Reference
Hack	2.5 [1.1–5.5], 0.02	4.3 [1.2–15.0], 0.02	9.0 [6.6–12.3], <0.001
Trot	0.8 [0.3–2.5], 0.7	3.3 [0.69–16.2], 0.1	2.6 [1.8–3.8], <0.001
Jumping	3.4 [1.7–6.5], 0.001	5.8 [1.9–17.8], 0.002	5.2 [4–6.9], <0.001
XC school	3.7 [1.4–9.6], 0.007	11.5 [3.0–44.4], <0.001	3.2 [2.0–5.0], <0.001
Gallop	4.7 [2.6–8.4], <0.001	6.6 [2.4–18.4], 0.001	2.7 [2.1–3.6], <0.001
Competition	11.4 [3.6–35.95], <0.001	13.5 [2.8–64.0], 0.001	3.6 [2.5–5.1], <0.001

## 4 DISCUSSION

It was feasible to obtain exercising ECGs (73.6% readable), and the workflow using mobile health technology allowed the collection and analysis of a large data set. The percentage of readable ECGs is lower in this study when compared with studies in which veterinarians obtained exercising ECGs in standardised exercise tests using the same fitness tracker^14^ (94%) or a different telemetry unit^7^ (95%) and similar to the percentage reported during cross‐country competition in a recent report using a telemetry unit (73%).^6^ The fact that operators did not have medical training in the study we present here, the different types of exercise recorded and the variable definition for a readable ECG need to be considered when comparing the percentage of readable ECGs between studies. It is likely that personnel with more training could obtain a higher percentage of readable ECGs using the same equipment, but the sample size of the study would not have been possible with the existing resources. The collection of exercising ECGs integrated into the monitoring of equine athletes could allow the analysis of large data sets potentially useful in adapting the concept of screening programs to decrease the frequency of arrhythmias that could predispose to performance problems or sudden cardiac death.^18^ The types and numbers of arrhythmias that may affect safety or performance, as well as automatisation of ECG analysis, are needed to implement a screening program concept in large populations of horses.

Interestingly, the percentage of readable recordings during Gallops and Jumping exercise (exercise with higher HR_peak_ and that include jumps) had a higher percentage of readable recordings than exercise recordings during ‘Flat’ workouts. Possible explanations include the motivation of the riders to collect information that they perceived as useful for the management of their athletes, more careful placement of tack for a high‐stakes situation or performing exercise in which sweat may be more likely to act as a coupling agent. Eventers do not exercise at maximal intensity/speed, are often clipped, and typically have a lean phenotype. These factors are perceived by the authors as potentially affecting ECG quality, and for this reason, the results about the readability of ECGs in this study cannot be extrapolated to other populations of horses.

The presence of arrhythmias was common in Eventing horses exercising with and without previous signs of cardiac disease. This is a frequently described finding in eventers and other equine athletes during training, exercise testing^6^ and competition^7^ in all types of equestrian activities^1^, ^2^, ^3^, ^4^, ^5^, ^6^, ^7^ as well as in fit human athletes.^19^ Complex arrhythmias were rare and complex arrhythmias during the recovery period were less common than described in studies reporting maximal intensity exercise.^2^, ^8^ It is possible that differences in intensity are the cause of this discrepancy. The results support previous literature that suggests that exercising arrhythmias are common, but also support that some types of arrhythmias are not common and should not be disregarded.

Arrhythmias were more frequent in horses with previous signs of cardiac disease. This was expected and may reflect the general principle that a substrate and a trigger are necessary to generate arrhythmias. It is possible that horses in the CARDIAC group were more likely to have an underlying substrate for arrhythmogenesis that generated arrhythmias more frequently when exposed to the same or similar triggers. Despite the finding that more frequent arrhythmias were recorded in horses with previous signs of cardiac disease, none of the horses in the study group had reported performance problems or effects on the safety of the riders. It is interesting that one horse without previous signs of disease developed paroxysms of atrial fibrillation (AF). At the time of writing the manuscript, the horse that had developed paroxysms of AF had been monitored for 17 months without recurrence. In the 14 ECGs obtained before paroxysmal AF (12 gallops, 1 competition and 1 flat), there were occasional PCs except for the gallop prior to the first AF episode in which the NPC increased to 47 (previously maximum of 5 single PCs/workout), and there was a couplet and a triplet. Interestingly the rider of this horse did not detect abnormal behaviour or performance during the gallops when the horse developed paroxysmal AF despite the disproportionate sudden increase in heart rate and a simultaneous decrease in speed, stride rate and stride length showed by the fitness tracker (Figure 4). A horse with underlying myocardial disease and arrhythmias in which high‐intensity exercise was discouraged, following recommendation in a current expert consensus,^10^ never demonstrated clinical signs during the 49 months of monitoring. This individual displayed complex arrhythmias in 5 recordings of the 59 readable ECGs evaluated. This study and the contrasting case examples described add to the body of literature suggesting that arrhythmias that may affect safety or performance require better understanding.

Cardiac remodelling that occurs with endurance training (athlete's heart) and the physiological changes that accompany intense exercise (electrolyte disturbances, acid–base derangements and/or hypoxia) are factors for arrhythmogenesis in different settings.^20^, ^21^ However, we did not find age, level, or period of the season to be significantly associated with outcomes in the final multivariable logistic regression model in this study, despite initial associations found in exploratory univariate analysis. There was an association between HR_peak_ and different arrhythmia categories, like the described in previous reports.^1^, ^6^ It is interesting that in a previous study comparing training and competition heart rates in Eventing horses,^22^ heart rates recorded during training sessions were consistently much lower (138 ± 17/min) than during competition (195 ± 8/min). In the current study values of HR_peak_ during gallops were closer to competition values and overlapping in many instances. This may be the consequence of the different methodologies to select workouts or a better recognition of the relationship between competition demands and training stimulus by the riders in the current study. The collinearity of HR_peak_ and duration with the type of exercise did not allow the inclusion of these variables in final regression models. The goal of the current clinical study was to monitor horses during their usual training routines; therefore, the investigators did not control for duration or intensity. A different study design would be needed to separate these effects.

Circumstances surrounding exercise, the type of exercise test and familiarity with the test have also been suggested to affect arrhythmogenesis in horses and humans,^8^, ^9^, ^23^ potentially due to imbalanced sympathetically mediated excitation and parasympathetic withdrawal destabilising cardiac electrophysiology.^24^ Certain types of exercise were associated with an increased risk of arrhythmias but this variable was colinear with the heart rate, suggesting the intensity of exercise may be a key factor responsible for this association. It is interesting that arrhythmias were still frequent when exercise was performed following standard routines and with familiar environment and personnel, although a direct comparison with other conditions cannot be performed with the current study design.

Limitations of the study include the use of single lead exercising ECGs, which does not allow a determination of the origin or precise description of arrhythmias. Also, a single reader analysed all electrocardiograms and there is variability in the interpretation of arrhythmias.^25^ Finally, horses included in the NON‐CARDIAC group did not have complete cardiac evaluations and some may have had underlying cardiovascular disease. Due to these limitations and to accomplish the study goals, we elected to perform statistics on a limited number of variables. Triplets were considered as a complex arrhythmia and couplets as a non‐complex arrhythmia following recommendations for ‘Eligibility and Disqualification Recommendations for Competitive (human) Athletes with Cardiovascular Abnormalities’ written by the American Heart Association and American College of Cardiology.^11^ This should be considered when comparing results with other studies that may have used different definitions. We acknowledge that obtaining ECGs with more leads and additional sub‐classifications would provide additional information.

## 5 CONCLUSIONS

Arrhythmias were frequent in Eventing horses during training and competition, but complex arrhythmias were rare. The presence of previous signs of cardiac disease and type of workout affected the frequency of arrhythmias. The specific arrhythmia types and circumstances that should raise concern about performance and safety remain a relevant question for equine practitioners. This will require further studies evaluating large populations of horses, including horses that develop clinical signs.

## AUTHOR CONTRIBUTIONS

**Cristobal Navas de Solis:** Conceptualization; investigation; funding acquisition; writing – original draft; methodology; writing – review and editing; project administration; supervision. **Claire Solomon:** Investigation; data curation; writing – review and editing; formal analysis. **Mary Durando:** Conceptualization; methodology; writing – review and editing. **Darko Stefanovski:** Conceptualization; methodology; software; formal analysis; data curation; writing – review and editing.

## FUNDING INFORMATION

This study was supported by The Thomas B. McCabe and Jeannette E. Laws McCabe Fund.

## CONFLICT OF INTEREST STATEMENT

The authors declare no conflicts of interest.

## DATA INTEGRITY STATEMENT

Cristobal Navas de Solis had full access to all the data in the study and takes responsibility for the integrity of the data and the accuracy of the data analysis.

## ETHICAL ANIMAL RESEARCH

Institutional Animal Care and Use permission and Privately Owned Animal Consent Protocols were approved (IACUC806975).

## INFORMED CONSENT

Owners gave consent for their animals' inclusion in the study.

### PEER REVIEW

The peer review history for this article is available at https://www.webofscience.com/api/gateway/wos/peer-review/10.1111/evj.14449.

## Data Availability

The data that support findings of this study are available from the corresponding author upon reasonable request: Open sharing exemption granted by the editor due to lack of provision in the owner informed consent process.
